# Immunochemistry-Based Diagnosis of Extrapulmonary Tuberculosis: A Strategy for Large-Scale Production of MPT64-Antibodies for Use in the MPT64 Antigen Detection Test

**DOI:** 10.3390/antib10030034

**Published:** 2021-08-26

**Authors:** Ida Marie Hoel, Iman A Mohammed Ali, Sheeba Ishtiaq, Lisbet Sviland, Harald Wiker, Tehmina Mustafa

**Affiliations:** 1 Centre for International Health, Department of Global Public Health and Primary Care, University of Bergen, 5020 Bergen, Norway; mohammedaliiman@yahoo.com (I.A.M.A.); tehmina.mustafa@uib.no (T.M.); 2 Department of Clinical Science, University of Bergen, 5020 Bergen, Norway; harald.wiker@uib.no; 3 Department of Clinical Medicine, University of Bergen, 5020 Bergen, Norway; lisbet.sviland@helse-bergen.no; 4 Department of Histopathology, Gulab Devi Chest Hospital Lahore, Lahore 54000, Pakistan; sheeba_i@yahoo.com; 5 Department of Pathology, Haukeland University Hospital, 5021 Bergen, Norway; 6 Department of Thoracic Medicine, Haukeland University Hospital, 5021 Bergen, Norway

**Keywords:** extrapulmonary tuberculosis, diagnostics, antigen detection, MPT64, immunohistochemistry, polyclonal antibody, monoclonal antibody

## Abstract

Tuberculosis (TB) is a global health problem. The immunohistochemistry (IHC)-based MPT64 antigen detection test has shown promising results for diagnosing extrapulmonary TB in previous studies. However, the anti-MPT64 antibody currently used in the test is in limited supply, and reproduction of a functional antibody is a prerequisite for further large-scale use. Various antigen-adjuvant combinations and immunisation protocols were tested in mice and rabbits to generate monoclonal and polyclonal antibodies. Antibodies were screened in IHC, and the final new antibody was validated on clinical human specimens. We were not able to generate monoclonal antibodies that were functional in IHC, but we obtained multiple functional polyclonal antibodies through careful selection of antigen-adjuvant and comprehensive screening in IHC of both pre-immune sera and antisera. To overcome the limitation of batch-to-batch variability with polyclonal antibodies, the best performing individual polyclonal antibodies were pooled to one final large-volume new anti-MPT64 antibody. The sensitivity of the new antibody was in the same range as the reference antibody, while the specificity was somewhat reduced. Our results suggest that it possible to reproduce a large-volume functional polyclonal antibody with stable performance, thereby securing stable supplies and reproducibility of the MPT64 test, albeit further validation remains to be done.

## 1. Introduction

Tuberculosis (TB) is a global health problem with an estimated 10 million new cases and 1.4 million deaths in 2019 [1]. Approximately one-third of the estimated new TB cases each year are not diagnosed or reported. Extrapulmonary TB (EPTB), which is more common in children and people with HIV [2,3,4,5,6], poses a special diagnostic challenge due to the paucibacillary nature of the disease. This leads to variable and generally low sensitivity of routine microscopy, PCR and culture [1,7], and new improved diagnostic tests are needed. Tests based on the detection of mycobacterial antigens are of special interest, as they have the potential to provide rapid and direct evidence of active TB disease [8]. Two antigen-detection tests for TB diagnosis are currently commercially available (Alere Determine TB LAM and Fujifilm SILVAMP TB LAM, both detecting lipoarabinomannan in urine) but their clinical use is restricted due to suboptimal sensitivity [9,10]. The novel MPT64 antigen-detection test (the MPT64 test) for the diagnosis of EPTB, which is based on detection of the mycobacterial antigen MPT64 in tissue samples from the site of infection, has shown promising results in previous validation studies [11,12,13,14,15,16,17,18], with higher sensitivity than routine smear and culture in high TB incidence settings [12,13,14,15,16,17]. The test is feasible to implement in the low-resource setting and can contribute towards timely and accurate diagnosis of EPTB [16]. These results warrant further research to evaluate the diagnostic test accuracy in larger cohorts and to investigate the potential for large-scale use and clinical roll-out of the test. However, the in-house polyclonal rabbit anti-MPT64 antibody used in the test is currently available in limited amounts, and reproduction of an antibody with stable performance, which is a pre-requisite for large-scale use, can be challenging due to batch-to-batch to variations in polyclonal antibodies [19]. The aim of the study was to develop an anti-MPT64 antibody to secure stable supplies and performance of the MPT64 test. Here, we describe different aspects to be considered when developing antibodies for use in immunohistochemistry (IHC), the challenges faced with the production of a monoclonal antibody and strategies to make large volumes of a functional polyclonal antibody.

## 2. Materials and Methods

### 2.1. Production of MPT64 Antigen

Several strategies were used to produce MPT64 antigen for immunisation. Native MPT64 was produced because the conformational epitopes, which may be important targets in IHC, are conserved in native proteins. Native MPB64 protein was obtained from cultures of *Mycobacterium bovis* bacillus Calmette-Guérin Moreau (BCG Moreau), according to previously developed protocols for culturing and purification [20,21,22], with some modifications (Supplementary Materials. MPB64 is homologous to the *M. tuberculosis*-derived MPT64 protein used as an antigen when the reference anti-MPT64 antibody was generated [23], and the two proteins are hereafter collectively referred to as MPT64. Because production of native MPT64 is time-consuming due to the slow growth of the bacilli and the resulting low yield of MPT64 protein, we also produced recombinant MPT64 antigen in several expression systems. In our laboratory, untagged recombinant MPT64 protein was expressed in the non-pathogenic, fast-growing *M. smegmatis* mc^2^ 155 [24] transformed with the mycobacterial plasmid (pUV15tetORm [25]) modified to contain the mpb64 gene with its predicted secretion signal sequence (GenBank Accession No. AM412059.2; BCGM locus 1981c), according to previously developed protocols [25,26,27] (Supplementary Materials, Figure S1). His-tagged recombinant MPT64 was produced in *E. coli* (by Trenzyme Life Science Services, Konstanz, Germany) and in a mammalian cell line (by InVivo Biotech Services, Berlin, Germany), based on the MPT64 amino acid sequence from *M. bovis* BCG Moreau without the signal sequence (Supplementary Materials). Table 1 shows the predicted amino acid sequence of the different MPT64 proteins that were used as antigens in the study.

### 2.2. Development of a Monoclonal Anti-MPT64 Antibody

**Figure 1:**
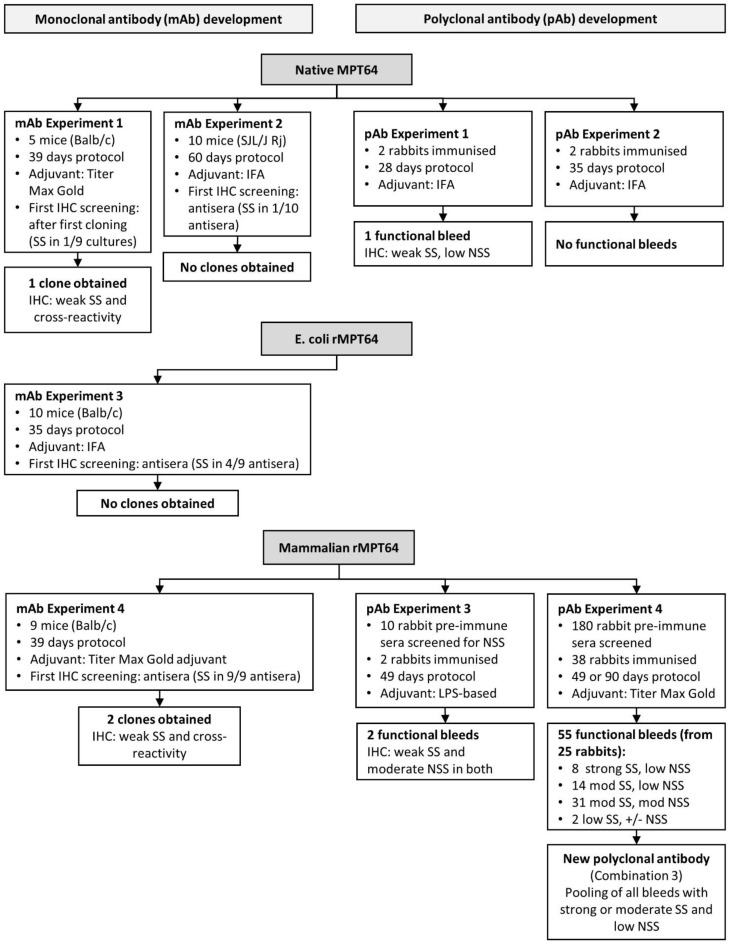
Overview of the different strategies used to develop monoclonal and polyclonal anti-MPT64 antibodies. Abbreviations; IFA, incomplete Freund adjuvant, IHC, immunohistochemistry; mod, moderate; NSS, non-specific staining; SS, specific staining; rMPT64, recombinant MPT64.

**Figure 2:**
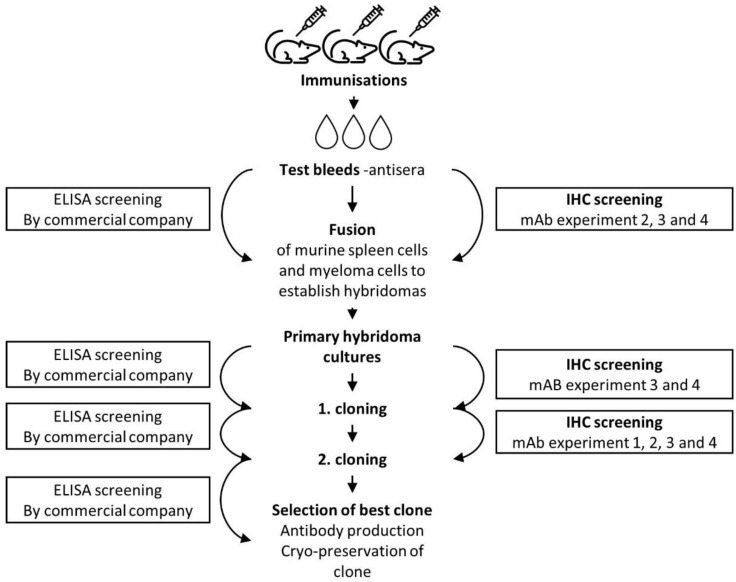
The steps of hybridoma production and different target points for screening of clones during the development of monoclonal antibodies. Abbreviations: ELISA, enzyme-linked immunosorbent assay, IHC, immunohistochemistry; mAb, monoclonal antibody.

Monoclonal antibodies from mice were generated by hybridoma technology according to standard methods [28] by commercial companies (Biogenes, Berlin, Germany; PharmAbs, Leuven, Belgium and InVivo BioTech Services, Hennigsdorf, Germany). Four different strategies to develop a functional monoclonal antibody for the MPT64 test were investigated (mAb experiment 1–4, Figure 1). Different combinations of MPT64 antigen and adjuvants were tested, and an increasing number of screening steps in IHC were added in subsequent experiments. Antibodies were screened in parallel in indirect enzyme-linked immunosorbent assay (ELISA) by the commercial companies, and in IHC at our laboratory, to identify the hybridomas that produced MPT64-specific antibodies. Antibody performance in IHC was assessed in positive and negative control tissue sections using serial dilution to find the optimal working dilution, by several readers (T.M. and I.M.H.). Several antigen retrieval methods were tested to optimise the MPT64 test protocol for murine monoclonal antibodies (Supplementary Materials). Figure 2 provides an overview of the different stages of hybridoma production and target points for screening of clones for monoclonal antibodies.

### 2.3. Development of Polyclonal Rabbit Anti-MPT64 Antibodies

#### 2.3.1. Immunisations

**Figure 3:**
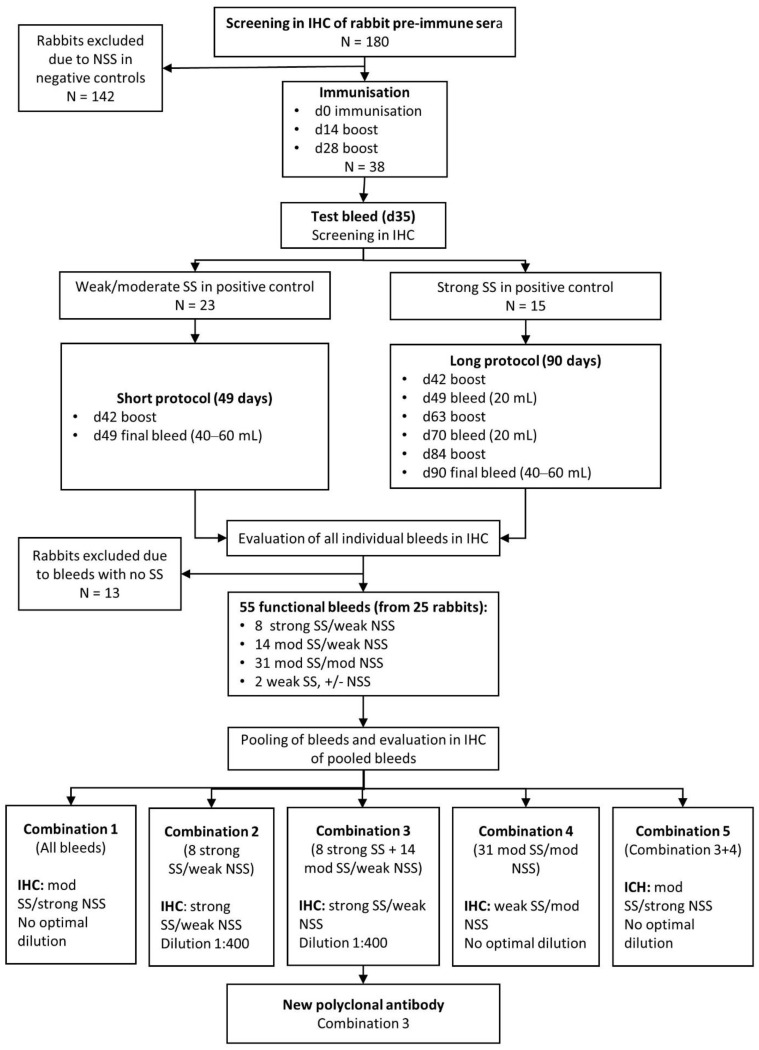
Flow chart showing immunisation protocols, screening and pooling strategies used to develop the new polyclonal antibody (polyclonal antibody experiment 4). Abbreviations: d, day; IHC, immunohistochemistry; mod, moderate; NSS, non-specific staining; SS, specific staining.

Immunisation of rabbits to produce polyclonal anti-MPT64 antibodies was performed by commercial companies (Biogenes, PharmAbs and InVivo). Figure 1 shows the four different strategies for development of polyclonal MPT64-antibodies that were investigated (pAb experiment 1–4). In experiments 1 and 2, the original protocol for development of the reference anti-MPT64 antibody was followed [29], with the exception that the antigen was not immunoprecipitated with polyclonal rabbit anti-MPT64 antibodies before immunisation. In brief, outbred female rabbits were immunised intradermally with native MPT64 emulsified in incomplete Freund adjuvant (IFA), using a standard immunisation protocol. In later experiments, recombinant MPT64 and other adjuvants were also tested; rabbits whose pre-immune sera gave non-specific staining were excluded from immunisation, and the immunisation protocols were longer for rabbits whose antisera tests bleeds gave particularly strong specific staining (Figure 3). In all experiments, pre-immune sera were collected at baseline, antisera test bleeds were collected seven days after the second or third immunisation and the final bleed was taken seven days after the last immunisation. Pre-immune sera and all individual bleeds were tested by indirect ELISA by the companies, and in IHC in our laboratory on positive and negative control tissue sections. The staining in IHC was evaluated by several readers (T.M., I.A.M.A. and I.M.H.). The optimal working dilution was determined using serial dilution.

#### 2.3.2. Selection and Pooling of MPT64-Specific Antibodies

The performance of the different bleeds in IHC were evaluated according to previously developed guidelines for interpretation of the MPT64 test [16]. The antibodies were categorised as (1) strong specific staining (SS) and low non-specific staining (NSS), (2) moderate SS and low NSS, (3) moderate to strong SS and moderate to strong NSS and (4) not functional, defined as no or weak SS and various degrees of NSS. In order to reduce batch-to-batch variation and to enable large-scale use of the polyclonal antibodies, the bleeds were pooled. Different combinations of pooled bleeds were tested in IHC (Figure 3), and the combination of bleeds with the best sensitivity and specificity in IHC, hereafter referred to as the new polyclonal antibody, was chosen for further experiments.

### 2.4. Background Blocking and Antibody Absorption Experiments

Blocking experiments were performed to reduce the non-specific binding of the new polyclonal antibody. Before application of the MPT64 antibody, the tissue sections were incubated with blocking solutions containing either (1) bovine serum albumin and serum free protein block with casein (Dako, Agilent), (2) normal goat serum or (3) recombinant Fc domain protein (Hu Fc block pure, BD, Becton Dickinson). Table 2 provides an overview of the different dilutions, incubation times and combinations of blocking solutions that were tested. Experiments with negative absorption were carried out at our laboratory by mixing the new polyclonal antibody with different proteinaceous solutions to allow non-specific antibodies or antibodies with cross-reactivity to bind proteins in the solutions and precipitate (Supplementary Materials). Positive purification by affinity column chromatography was also tested at an early stage of the study on one of the best-performing individual polyclonal antibodies, but resulted in a loss of specific staining in IHC, and the method was, therefore, not further explored.

### 2.5. Immunohistochemistry (the MPT64 Test)

The MPT64 test was performed using the Dako Envision + System-HRP kit (Agilent, Santa Clara, CA, USA), according to the manufacturer’s protocol, with some modifications. Briefly, 4 µm thick tissue sections on Superfrost Plus slides (Thermo Fisher Scientific, Waltham, MA, USA) were deparaffinised with xylene and rehydrated through decreasing grades of alcohol. When rabbit antibodies were used as primary antibody, heat-induced antigen retrieval (HIER) was performed by microwave boiling the sections in citrate buffer at pH 9, for 20 min. For murine antibodies, HIER was performed by pressure cooker boiling at 125 °C in TE-buffer at pH 9, for 1 min. The sections were left to cool for 20 min at RT, washed in distilled water for 10 min and incubated with peroxidase block for 20 min. For IHC with the new polyclonal antibody, an additional protein blocking step was then added to the protocol, in which a combination of 10% normal goat serum and 3% bovine serum albumin was applied to the sections overnight at 4 °C, followed by a serum-free protein block (Dako) for 12 min at RT the next day. The primary antibody was applied, and the slides were incubated for 60 min, before horseradish peroxidase conjugated secondary anti-rabbit antibody was applied for 45 min. Thereafter, the substrate (3-amino-9-ethylcarbazol) was added to the slides for 15 min, followed by counterstaining with Mayer’s haematoxylin and mounting with Immu-Mount (Thermo Fisher Scientific, Waltham, MA, USA). Slides were washed with wash buffer (0.05 mol/L Tris/HCl buffered saline with 0.05% Tween 20, pH 7.6) between all incubation steps. In each IHC run, tissue sections from known TB and non-TB cases were added as controls. In addition, the primary antibody was substituted with antibody diluent on one non-TB tissue section, to assess any non-specific binding of the secondary antibody or other reagents during IHC.

### 2.6. Validation of the New Polyclonal Antibodies

Human clinical samples were used for the validation of the new anti-MPT64 polyclonal antibody. Twenty extrapulmonary biopsies from TB cases with a confirmed (culture and/or Xpert MTB/RIF positive) or clinical TB diagnosis (defined as patients with presumptive extrapulmonary TB, and histology suggestive of TB, and response to treatment, as assessed by the primary investigator) were used. These materials were collected as part of another project where the clinical samples were obtained from a cohort of EPTB patients [30]. Twenty-four non-TB samples with histopathological diagnoses other than TB were used as controls. The immunostaining was screened at a total magnification of 200× and evaluated in detail at 400× by one designated reader (S.I.) according to previously developed guidelines for interpretation of the MPT64 test [16]. The reader was blinded to the TB status of the samples. Briefly, a sample was positive if a minimum of two granular red-brown coloured spots, either observed intracytoplasmic in inflammatory cells or extracellularly in necrotic material, were present in the sample. No staining, nuclear staining or extracellular granular staining in non-necrotic areas were interpreted as negative.

### 2.7. Statistical Methods

The sensitivity and specificity of the new polyclonal antibody were calculated using 2 × 2 cross-tabulation against a reference standard that included culture, Xpert MTB/RIF and clinical TB diagnosis.

### 2.8. Ethical Considerations

All animal experiments were performed by commercial companies that are certified according to ISO 9001. The animal keeping and corresponding works were performed according to German/Belgian country specific, European and US NIH/OLAW guidelines. The human biopsies used for the validation of the test were used from a study approved by the National Bioethics Committee of Pakistan (Islamabad, Pakistan) and the Regional Committee for Medical and Health Research Ethics of Western Norway (REK vest) (ethical approval code: 2014/46/REK vest). A written informed consent was obtained from all the participants in the study.

## 3. Results

### 3.1. Production of MPT64 Antigen

During a period of one year, 156 litres of BCG Moreau culture filtrates were produced and subsequently purified by chromatography (Figure S2), resulting in a yield of approximately 15 mg MPT64 protein with a purity of >90%, based on visual evaluation of Coomassie-stained SDS-PAGE gels (Figure S3). Untagged recombinant MPT64 was expressed in *M. smegmatis* at our laboratory, and the presence of MPT64 in the cultures was confirmed by a positive MGIT TBc identification test. No band of the expected size of MPT64 was found on Coomassie stained SDS-PAGE gel, neither from the concentrated culture filtrate nor cell lysate solution, but both solutions gave a band of the right size in western blot. This indicated that soluble MPT64 had been expressed, but in low quantities. Purified HIS-tagged recombinant MPT64 protein from *E. coli* (Trenzyme) and mammalian HEK-cells (In Vivo) were provided from commercial companies.

### 3.2. Development of Monoclonal MPT64 Antibody

Figure 1 provides the results from the four strategies that were tested to develop a monoclonal anti-MPT64 antibody. Monoclonal antibodies that were functional in ELISA were obtained with all the strategies, but most of these antibodies gave no specific staining in IHC. As ELISA was not suitable to select clones that were functional in IHC, we included earlier and more frequent screening in IHC in the latter experiments (Figure 1 and Figure 2). In experiment 2–4, only mice whose antisera showed strong SS in IHC were used for fusion, and we observed that the combination of mammalian recombinant MPT64 and Titer Max Gold adjuvant resulted in particularly good polyclonal antisera in the mice (SS in 9/9 mice), as compared to native MPT64 and IFA (SS in 1/10 mice), or *E. coli* recombinant MPT64 and IFA (SS in 4/9 mice). However, hybridoma cultures from mice with particularly good antisera did not result in more SS on IHC despite very good reactivity on ELISA. The few clones that gave possible SS with IHC also displayed cross-reactivity, and development of a monoclonal antibody was not further pursued after the fourth experiment.

### 3.3. Development of a Polyclonal Antibody

**Figure 4:**
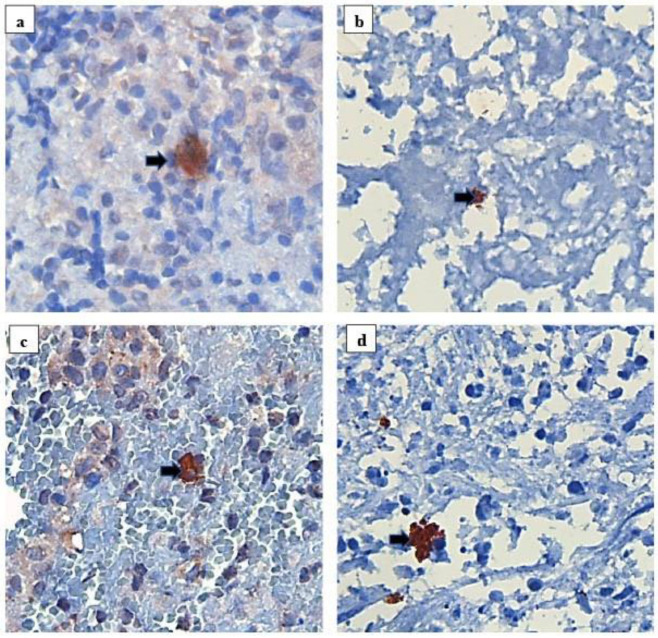
Immunohistochemistry with the new (**a**,**c**) and reference anti-MPT64 polyclonal antibody (**b**,**d**) on tissue sections from patients with tuberculosis lymph adenitis. Specific staining is seen as reddish-brown granular staining (arrow) located intracellularly in the lesions. The weak diffuse staining in a and c is non-specific staining.

The results from the four strategies used to develop a polyclonal antibody are summarised in Figure 1. Based on the results from experiment 1–2, where non-specific staining and weak specific staining were observed in the majority of antibodies, the strategy of screening was modified in experiment 3–4 (Figure 1 and Figure 4). To minimise non-specific binding, we adopted a strategy of only selecting the rabbits whose pre-immune sera showed minimal or no staining in IHC for further immunisations. Additionally, the adjuvant was changed to Titer Max Gold together with mammalian recombinant MPT64 as antigen in experiment 4, as particularly good polyclonal antisera had been obtained with this combination in mice during development of monoclonal antibodies. Furthermore, the rabbits whose antisera gave particularly strong specific staining in IHC in experiment 4, were selected for a longer immunisation protocol (90 days) to generate larger volumes of antisera (Figure 3). Using this strategy, a total of 38 rabbits were immunised in experiment 4 (whereas 142 rabbits were excluded after screening of pre-immune sera and released for other projects within the company), resulting in 55 bleeds from 25 animals that were functional in IHC (Figure 3). These bleeds were further pooled in different combinations (combination 1–5), to increase the total antibody volume and reduce the batch-to-batch variation. Combination 2 and 3 gave the best results in IHC, both with equal performance, showing strong SS that was comparable to the reference antibody, and weak NSS (Figure 4). Combination 3, which included all individual bleeds with good SS (defined as strong or moderate SS) and low NSS, was chosen as the new polyclonal antibody because of the larger volume as compared to combination 2, making it suitable for future large-scale use. Among the various strategies employed to reduce NSS in the new polyclonal antibody, blocking with bovine serum albumin 3% and normal goat serum 10% overnight followed by serum-free block for 12 min gave the best results with clearly reduced NSS (Table 2). This blocking step was incorporated into the MPT64 test protocol for the new polyclonal antibody. Negative absorption of the new antibody with different proteinaceous solutions, including *M. bovis* BCG Copenhagen components, did not reduce NSS.

### 3.4. Validation of the New Polyclonal Antibody

Validation of the new polyclonal antibody was performed on human clinical extrapulmonary specimens, including 20 biopsies from confirmed or clinically diagnosed TB cases and 24 biopsies from non-TB cases. In the TB samples, the sensitivity of ZN, Xpert, Culture and the new polyclonal MPT64 antibody was 11% (1/9), 67% (6/9), 40% (6/15) and 95% (19/20), respectively (Table 3). The new polyclonal antibody was positive in all the culture and/or Xpert positive TB samples (*n* = 12), and positive in 7/8 samples from clinically diagnosed TB cases. Non-specific staining was observed in 4/24 non-TB samples with the new polyclonal antibody, yielding an overall specificity of 83%.

## 4. Discussion

In this study, we have investigated different strategies to develop a new functional antibody for use in the TB diagnostic MPT64 test, which has shown promising results for diagnosing EPTB in low-resource settings [11,12,13,14,15,16,17]. The test uses an in-house polyclonal antibody for the detection of the mycobacterial antigen MPT64, but the antibody is in limited supply and further large-scale use of the test requires reproduction of the antibody. Despite generation of several monoclonal anti-MPT64 antibodies with good reactivity in ELISA, none of the antibodies were fully functional in IHC. We, therefore, opted for the development of a polyclonal antibody. By careful selection of animals for immunisation, optimal antigen-adjuvant combination, screening of antibodies in IHC and pooling of the best performing individual antibodies, the generation of a sensitive new polyclonal antibody in a large volume was achieved. The new antibody was more sensitive than routine microscopy, Xpert and culture when validated on a small number of clinical samples, albeit a reduced specificity warrants more work with background reduction.

The choice of antigen and adjuvant can greatly affect the performance of the resulting antibody [31,32,33,34]. We used native MPT64 as antigen in the initial experiments, because the conformational epitopes, which may be important targets in IHC [35], are conserved in native proteins. However, as the resulting antibodies were neither sensitive nor specific in IHC, and the production of native MPT64 was time-consuming, we changed to recombinant MPT64 in the latter experiments. Recombinant protein expression allows for rapid production but may alter conformational epitopes depending on the choice of host system, partly because of differences in post-translational modification systems between species. Recombinant MPT64 from *E. coli* has been reported to elicit weaker immune responses than native MPT64 and recombinant MPT64 from *M. smegmatis* [36], possibly due to different post-translational modification systems [37,38,39,40,41,42,43], suggesting that important MPT64 epitopes can be affected by the host system. Despite this possible drawback, immunisation of mice with recombinant MPT64 from both *E. coli* and mammalian cells resulted in polyclonal antisera with as strong, or stronger, specific staining in IHC as compared to immunisation with native MPT64 (strong specific staining was observed in 9/9, 4/9 and 1/10 murine antisera with mammalian MPT64, *E. coli* MPT64 and native MPT64 as antigen, respectively). Poor quality of the native MPT64 antigen, possibly due to misfolding of the protein, could be one reason for the low performance of the resulting antibodies, but this was not further investigated. Several adjuvants, which can enhance and prolong the immune response [33,34], were also tested in the study (Figure 2). Based on the particularly strong specific staining obtained after immunisation of mice with mammalian recombinant MPT64 and Titer Max Gold adjuvant, we chose to change to this antigen-adjuvant combination in rabbits as well. This resulted in some of the best individual antisera in our study, with almost as strong specific staining as the reference antibody, and low non-specific staining. Furthermore, the dose of antigen injected may also impact the immune response and antibodies generated [33], but this was not explored in our study, as all rabbits received the same dose of antigen.

The main reason for choosing monoclonal antibodies in a diagnostic test is to avoid batch-to-batch variability, thereby securing reproducible test performance. Still, polyclonal antibodies offer some important advantages [19]. The development of polyclonal antibodies is relatively simple, fast and inexpensive and can result in highly sensitive antibodies because several epitopes are recognised simultaneously, leading to efficient signal amplification and improved detection. High test sensitivity is of great importance in TB diagnostics, as the sensitivity of routine TB diagnostic tests is low in paucibacillary EPTB disease [44]. Furthermore, the biological diversity of polyclonal antibodies allows for use under a wide range of chemical conditions and temperatures, which is an advantage in low-resource settings. Thus, as long as batch-to-batch consistency is managed through standardised validation of all new batches, or through creation of a large batch as in this study, polyclonal antibodies may be used, and are being used, for diagnostic purposes [45,46,47]. Still, cross-reactivity is a common issue with polyclonal antibodies, and antibody purification or background blocking is often required. Surprisingly, negative absorption with BCG Copenhagen culture filtrate proteins, which successfully reduced non-specific staining in the reference antibody, had no effect on the new polyclonal antibody. We experienced that a combined strategy of careful selection of animals for immunisation and optimised background blocking prior to IHC were the most effective measures to reduce non-specific staining. The specificity of the new antibody is still not optimal, but ongoing work indicates that increased duration of the washing steps in IHC removes the non-specific staining without reducing the specific staining of the antibody. This will be further explored in an upcoming validation study.

The development of monoclonal antibodies for use in IHC can be challenging, as demonstrated in our study. The screening of hybridoma cultures in ELISA was not an optimal method to select clones that are functional in IHC. Clones with high performance in ELISA may not detect relevant epitopes in IHC, because formalin fixation prior to IHC can mask or alter the three-dimensional conformation of the epitopes that were exposed during ELISA [48]. Antigen retrieval partly reverses these alterations, but the effect varies between epitopes, and antibody screening should, therefore, preferably be performed directly in IHC. This was done in the latter experiments, but is more time-consuming than ELISA and requires large numbers of positive control tissue sections. In the latter experiments, antisera from several immunised mice gave relatively strong specific staining in IHC, but the resulting few monoclonal antibodies that showed some specific staining also displayed cross-reactivity, indicating that their target epitopes were not unique to the MPT64 protein. We may have lost clones that recognised unique MPT64 epitopes during fusion or hybridoma selection, especially in the initial experiments where IHC was not used for screening. Another possible explanation is that our choice of antigen was not optimal to generate antibodies against unique epitopes [49]. If the less unique epitopes are immunodominant, most of the antibodies in the mice will be generated against these, whereas less immunogenic, but unique epitopes could be missed by the immune system. To avoid this, so-called subtractive immunisation techniques can be applied, in which the undesirable antibodies are used to mask their epitopes on the antigen before immunisation, so that antibodies are only generated against other epitopes [49]. This remains to be explored during further development of monoclonal MPT64-antibodies for IHC.

## 5. Conclusions

Reproduction of a functional polyclonal MPT64 antibody for large-scale use of the TB diagnostic MPT64 test was achieved through a combination of careful selection of antigen-adjuvant for the immunisation protocol, comprehensive screening in IHC of both pre-immune sera and antisera to find the best performing antibodies, followed by pooling the best individual antisera to obtain a large volume of polyclonal antibodies with stable performance, thereby securing stable supplies and reproducibility of the MPT64 test. Further validation of the new polyclonal antibody in clinically relevant larger populations remains to be done.

## Figures and Tables

**Table 1:** Amino acid sequence of the various forms of MPT64 protein used as antigen in the study. The native MPT64 sequence is derived from *M. bovis* BCG Moreau and includes a N-terminal cleavable protein secretion signal sequence (bold), which is not present in the final, secreted form of the protein. The sequence of the recombinant protein produced in *Escherichia coli* (*E. coli* rMPT64) includes a C-terminal Thrombin-cleavable 8XHis-tag (underlined) to simplify purification. The sequence of the recombinant protein produced in human HEK cells (mammalian rMPT64) includes a N-terminal HSA signal peptide (bold) to secrete the protein, and a C-terminal 6XHis-tag (underlined) to simplify purification.

Protein	Amino Acid Sequence
Native MPT64	**MRIKIFMLVTAVVLLCCSGVATA**APKTYCEELKGTDTGQACQIQMSDPAYNINISLPSYYPDQKSLENYIAQTRDKFLSAATSSTPREAPYELNITSATYQSAIPPRGTQAVVLKVYQNAGGTHPTTTYKAFDWDQAYRKPITYDTLWQADTDPLPVVFPIVQGELSKQTGQQVSIAPNAGLDPVNYQNFAVTNDGVIFFFNPGELLPEAAGPTQVLVPRSAIDSMLA
*E. coli* rMPT64	MAPKTYCEELKGTDTGQACQIQMSDPAYNINISLPSYYPDQKSLENYIAQTRDKFLSAATSSTPREAPYELNITSATYQSAIPPRGTQAVVLKVYQNAGGTHPTTTYKAFDWDQAYRKPITYDTLWQADTDPLPVVFPIVQGELSKQTGQQVSIAPNAGLDPVNYQNFAVTNDGVIFFFNPGELLPEAAGPTQVLVPRSAIDSMLAVLVPRGSAAALEHHHHHHHH
Mammalian rMPT64	**MKWVTFISLLFLFSSAYS**APKTYCEELKGTDTGQACQIQMSDPAYNINISLPSYYPDQKSLENYIAQTRDKFLSAATSSTPREAPYELNITSATYQSAIPPRGTQAVVLKVYQNAGGTHPTTTYKAFDWDQAYRKPITYDTLWQADTDPLPVVFPIVQGELSKQTGQQVSIAPNAGLDPVNYQNFAVTNDGVIFFFNPGELLPEAAGPTQVLVPRSAIDSMLAHHHHHH

**Table 2:** Strategies employed to reduce non-specific staining in immunohistochemistry with the new polyclonal antibody.

	Level of Non-Specific Staining	
Strategy	Positive TB Control	Negative Non-TB Control
Blocking experiments		
Serum free block (12 min, 30 min, 60 min, or overnight)	-	-
BSA 3% or 10% and NGS 10% (60 min), followed by serum free block (60 min)	-/↓	-/↓
BSA 3% or 10% and NGS 10% (overnight), followed by serum free block (12 min)	↓↓	↓↓
Fc block	-	-
Absorption experiments		
*M. bovis* BCG Copenhagen, culture filtrates	-/↓	-/↓
*M. bovis* BCG Copenhagen, cell sonicate	-	-
Homogenised non-TB lung and lymph node tissue sections (deparaffinised and hydrated)	-	↑

Abbreviations: TB, tuberculosis; BSA, bovine serum albumin; NGS, normal goat serum; NSS, non-specific staining; SS, specific staining; *M. bovis*, *mycobacterium bovis*; (-), no change; (↑), increased; (↓), decreased.

**Table 3:** Results of routine diagnostic tests and the MPT64 test performed on clinical TB and non-TB samples.

		Routine Diagnostic Tests Positive/Total (%)			The MPT64 Test Positive/Total (%)
	*N*	Ziehl-Neelsen	Xpert MTB/RIF	Culture LJ	New Polyclonal MPT64 Antibody
TB cases total	20	1/9 (11)	6/9 (67)	6/15 (40)	19/20 (95)
Lymph node biopsies	14	1/6 (17)	3/4 (75)	6/12 (50)	14/14 (100)
Other biopsies	6	0/3 (0)	3/5 (60)	0/3 (0)	5/6 (83)
Confirmed TB cases	12	0/5 (0)	6/6 (100)	6/11 (55)	12/12 (100)
Clinically diagnosed TB cases	8	1/4 (25)	0/4 (0)	0/3 (0)	7/8 (88)
Non-TB cases total	24	N/A	N/A	N/A	4/24 (17)
Lymph node biopsies	7	N/A	N/A	N/A	2/7 (29)
Other biopsies	17	N/A	N/A	N/A	2/17 (12)

Abbreviations: LJ, Lowenstein Jensen; TB, tuberculosis.

## Data Availability

The data presented in this study are available on request from the corresponding author.

## Supplementary Materials

The following are available online at https://www.mdpi.com/article/10.3390/antib10030034/s1, Supplementary Materials: Detailed protocols for production and purification of MPT64 antigen, development of antibodies and immunohistochemistry background reduction, Figure S1: The completed vector construct, pUV15tetORmMpt64, expressing the MPT64 protein, Figure S2: Chromatograms from the three-step chromatography purification strategy applied to purify native MPT64 protein, Figure S3: Overview of the purity of the MPT64-containing fractions after each step of chromatography.
