# Supplementary material for: Immunochemistry-Based Diagnosis of Extrapulmonary Tuberculosis: A Strategy for Large-Scale Production of MPT64-Antibodies for Use in the MPT64 Antigen Detection Test
Source: Antibodies (Basel). 2021 Aug 26;10(3):34. doi: 10.3390/antib10030034 (PMC8406093; doi:10.3390/antib10030034)
Supplement: Supplementary file 1 [file antibodies-10-00034-s001.zip › antibodies-1302522-supplementary.pdf]

Supplementary materials to manuscript "Immunochemistry-based diagnosis of extrapulmonary tuberculosis: A strategy for large-scale production of MPT64-antibodies for use in the MPT64 antigen detection test"

Supplementary text

Detailed protocols for production and purification of MPT64 antigen, development of antibodies, and immunohistochemistry background reduction

Production of native MPT64 protein

The Brazilian BCG vaccine strain *M. bovis* bacillus Calmette-Guérin Moreau (BCG Moreau) was used for production of endogenous, native MPB64 protein. Briefly, lyophilised BCG cells were suspended in isotone saline and inoculated onto Lowenstein-Jensen slants and subsequently onto wholly synthetic Sauton's liquid medium to be grown as surface pellicles at 37°C without shaking. The cultures were harvested after three weeks, as the peak concentration of secreted MPT64 protein from *in vitro* grown *M. tuberculosis* is obtained at this time point (20). The bacilli were removed with paper filter from the culture filtrates which were sterile filtered (0.2 µm) before further use.

Purification of native MPT64 protein

The methods for protein purification were based on previous protocols (21, 22), with some modifications. To concentrate the proteins in the culture filtrates, solid ammonium sulphate was added to 80% saturation (533 g/L at 4°C). Proteins were left to precipitate overnight, followed by centrifugation at 10,000 x *g* at 4°C for 45 minutes. The protein pellet was resuspended in phosphate-buffered saline (PBS) and dialysed against PBS. Protein concentration was measured on Direct Detect Spectrometer (Merck Millipore, MA, USA).

In order to purify the untagged, native MPT64 protein (nMPT64) to the required level of purity for antibody production (80-90%), a three-step chromatography strategy, carried out under native conditions, including ion-exchange chromatography (IEX), hydrophobic interaction chromatography (HIC) and size exclusion chromatography (SEC), was used. All purification steps were performed on fast liquid protein chromatography (FPLC) machines (Äkta Explorer or

Purifier) at room temperature (RT), using filtered (0.45 µm) and degassed buffers and protein samples. The pH of all buffers was adjusted at RT.

Before the first step IEX, the protein concentrate was dialysed against IEX binding buffer (20 mM Tris-HCl, pH 8.3), and loaded onto a Q Sepharose Fast Flow XK 50/20 column that was subsequently washed with binding buffer until unbound proteins were removed. For elution, a linear gradient of 0 to 0.3 M NaCl in 20 mM Tris-HCl, pH 8.3, was applied, and 10 mL fractions of the eluate were consecutively collected. Between each chromatography step the collected fractions were subjected to SDS-PAGE under non-reducing conditions and Western blot with polyclonal rabbit anti-MPT64 antibody to assess purity and identify MPT64. MPT64 positive fractions were pooled and concentrated by ultrafiltration (Amicon Ultra spin-filter, molecular weight cut-off 3000, Merck, Germany) at 3200 x *g* for 25 minutes at RT. To prepare the sample for the intermediate HIC step, the sample buffer was exchanged to HIC binding buffer (20 mM Tris-HCl, 1.7 M ammonium sulphate, pH 7.5) by adding HIC binding buffer to the concentrated protein sample in the spin-filter, followed by ultrafiltration. This procedure was repeated twice. The protein sample was then loaded onto a Phenyl HP HiTrap column (1 mL bed volume) in HIC binding buffer. The column was washed with binding buffer until unbound proteins were removed, followed by elution of bound proteins by applying a linear gradient of 0-100% elution buffer (20 mM Tris-HCl, pH 7.5). 2 mL fractions of the eluate were collected during the run, and the fractions containing MPT64 were further pooled. Concentration and buffer exchange to SEC buffer (20 mM Tris-HCl, 0.3 M NaCl, pH 7.5) of the pooled protein sample were carried out by ultrafiltration as described above. For the final SEC polishing step, the protein sample was loaded onto a Superdex 75pg HiLoad 26/600 column in SEC buffer, separated according to molecular size as the sample passed through the medium over a volume of 400 mL SEC buffer at a flow rate of 2,5 mL/min, and collected as 5 mL fractions. The level of purity in the fractions containing MPT64 was visually evaluated in Coomassie stained SDS-PAGE gels. Fractions with a purity of approximately >90% were selected for later use as antigen. Endotoxin levels were measured (Pierce LAL chromogenic endotoxin quantitation kit, ThermoFisher Scientific) before and after endotoxin affinity purification (Pierce high capacity endotoxin removal spin columns, ThermoFisher Scientific). Protein concentration was measured by Direct Detect. The samples were stored at -80°C.

Production of recombinant MPT64 protein

Mycobacterial vector system

All laboratory kits were used according to the manufacturer's recommended protocols, unless otherwise stated. Genomic DNA from BCG Moreau was prepared using FastDNA SPIN Kit (MP Biomedicals, OH, USA). An 888 bp segment of genomic DNA containing the mpb64 gene with its

predicted secretion signal sequence (GenBank Accession No. AM412059.2; BCGM locus 1981c), was amplified using KAPA HiFi HotStart Readymix PCR kit (Roche) and the primers mpt64UP1 5'-ATCGCGGCAATCCAATCTCC-3' and mpt64LP1 3'-TCTCTAGCGACGATTCTTGAGC-5' (Initial denaturation 3 min 95°C. 30 amplification cycles: 20 sec 98°C, 15 sec 63°C, 20 sec 72°C. Final extension 1 min 72°C). Following purification with UltraClean 15 DNA Purification kit (MO BIO Laboratories), the PCR product was used as template to amplify an 853 bp segment containing the mpt64 coding region with PacI and PstI restriction sites, using the primers mpt64adaptUP1 5'-GCTTAATTAACTACTCCCGGAGGAA-3' and mpt64LP1 3'-TCTCTAGCGACGATTCTTGAGC-5' with the same PCR and purification protocols. The purified PCR product and the mycobacterial inducible plasmid pUV15tetORm were separately digested with PacI and PstI (New England BioLabs, MA, USA) and purified. Cloning was performed by incubating the plasmid backbone and insert (insert:backbone ratio 5:1), T4 DNA ligase and T4 DNA Ligase Buffer (Invitrogen) at 4°C overnight. Correct insertion was controlled by sequencing. Figure S1 shows the final plasmid construct and table 1 shows the predicted amino acid sequence of the expressed protein. Chemically competent E. coli cells (One Shot TOP10, ThermoFisher Scientific, MA, USA) were transformed with the plasmid by heat shock, plated on selective Luria Bertani (LB) agar, expanded in selective liquid LB medium (both containing 200 mg/L hygromycin (ThermoFisher Scientific)) and purified using QIAprep Spin Miniprep Kit (QIAGEN, Netherlands).

Electrocompetent M. smegmatis mc^2 155 was cultured in Middlebrook 7H9 supplemented with OADC, 0.05% Tween-80 and 0.2% glycerol, and prepared for transformation in glycerol as described by Goude and colleagues (26). The pUV15tetORmMpt64 plasmid was transformed into M. smegmatis mc^2 155 by electroporation (Bio-Rad Gene Pulse II) in a 0.2 cm gap electroporation cuvette subjected to one single pulse of 2,5kV, 25µF, with the pulse-controller resistance set at 1000Ω. After 3 hours cell recovery in 7H9 without antibiotics, the cells were harvested by centrifugation at 3000 x g for 10 minutes and plated out in suitable dilutions on

Middlebrook 7H10 agar plus 50 mg/L hygromycin. At day 3, transformant colonies were used to inoculate 5 mL 7H9 plus 50 µg/mL hygromycin and incubated on a shaker (100 rpm) at 37°C to reach logarithmic phase (OD_{600} 0.8-1). The culture was then inoculated into 200 mL 7H9 plus 50µg/mL hygromycin and 200ng/mL anhydrotetracycline, to induce expression of rMPT64 (27), and was incubated on a shaker (100 rpm) at 37°C for 3 days. Expression of rMPT64 was confirmed by subjecting 10µL of the culture diluted in 90 µL 7H9, to the MGIT TBc identification test (Becton Dickinson, NJ, USA), a rapid lateral-flow immunochromatographic assay normally used for detection of MPT64 antigen in MTBC cultures.

To assess if soluble rMPT64 had been expressed, the protein content in culture filtrates and cell sonicates were analysed. Culture filtrate was prepared from half of the culture as previously described. The other half was centrifuged at 3000 x *g* for 10 min at 4°C to pellet out bacterial cells. The bacteria were dissolved in PBS and probe sonicated in bursts of 30 sec over 10

minutes in a rosette cooling cell in an ice bath. The resulting cell lysate was centrifugated at 3000 x *g* for 10 min at 4°C and the supernatant was sterile filtered. Solid ammonium sulphate was added to 80% saturation (533 g/L at 4°C) to the culture filtrate and cell lysate supernatant and gently mixed by stirring. Proteins were left to precipitate overnight, followed by centrifugation at 10,000 x *g* at 4°C for 45 minutes. Each protein pellet was resuspended in 2 mL PBS, and 10 µL of the solution was analysed by SDS-PAGE and Western blot with polyclonal rabbit anti-MPT64 antibody to identify any rMPT64 protein bands.

To avoid protein contamination of the antigen from the culture medium, transformant colonies were also cultured in protein-free, wholly synthetic Sauton's medium instead of 7H9, otherwise using the same protocol as described above.

E. coli vector system

A recombinant MPT64-His protein was produced in an *E. coli* expression system (E. coli rMPT64) by Trenzyme Life Science Services (Konstanz, Germany). The E. coli rMPT64 was designed based on the MPT64 amino acid sequence from *M. bovis* BCG Moreau without the signal sequence and with a C-terminal 8xHis-tag (table 1). Cloning, transformation of *E. coli* and expression of the protein was performed according to standard protocols. The rMPT64 was purified under non-denaturing conditions using affinity chromatography, followed by dialysis against tris-buffered saline (TBS), pH 7.5. Purity (> 98%) was measured by densitometry of Coomassie stained SDS-PAGE gel. Endotoxin content was measured by LAL before and after endotoxin removal by affinity purification. The protein was stored at -80°C.

Mammalian cell line vector system

A recombinant MPT64 protein expressed in a human cell line (mammalian rMPT64) was produced by InVivo Biotech Services (Berlin, Germany). The mammalian rMPT64 was made based on the MPT64 amino acid sequence from *M. bovis* BCG Moreau without the signal sequence, with an HSA signal peptide at the N-terminal and a 6xHis-tag at the C-terminal (table 1) using an expression vector that was expanded in E. coli and then transfected into HEK cells, a cell line derived from human embryonic kidney cells, to express recombinant protein. Affinity chromatography was used to purify the rMPT64, followed by dialysis against TBS, pH 7.4. A purity of >90% was obtained, as determined by analysis of Coomassie-stained SDS-PAGE. Endotoxin levels were measured by LAL. The protein was stored at 2-8° C.

Development of a monoclonal and polyclonal anti-MPT64 antibody

Antigen retrieval experiments

Tissue sections were subjected to several antigen retrieval protocols prior to IHC to optimise the MPT64 test protocol for murine monoclonal antibodies. After deparaffinisation with xylene and

rehydration through decreasing grades of alcohol, the positive control tissue section were treated with 1) proteinase K digestion for 5 min, 2) heat induced epitope retrieval (HIER) in microwave oven in citrate buffer, pH 9, 20 min, 3) HIER in pressure cooker at 125°C in Tris-EDTA buffer, pH 9, for 1 min, and 4) no retrieval. The method that resulted in the strongest specific staining was used in further IHC experiments with monoclonal antibodies.

Background blocking experiments

In addition to the blocking of endogenous peroxidase, which is part of the original MPT64 test protocol, further blocking experiments were performed to reduce non-specific binding to tissue components when the new polyclonal antibody was used. Before application of the primary antibody, the tissue sections were incubated with different blocking solutions containing 1) proteins that bind readily to non-specific sites, including bovine serum albumin (BSA) and serum free protein block with casein (Dako, Agilent), 2) normal goat serum (NGS), in which antibodies in non-immune serum bind non-specific sites in the tissue, and 3) recombinant Fc domain protein (Hu Fc block pure, BD, Becton Dickinson) to block Fc receptors on immune cells in the tissue sections which could otherwise bind the Fc domain of the primary antibodies. Table 2 provides an overview of the different dilutions, incubation times and combinations of blocking solutions that were tested.

Absorption experiments

Experiments with negative absorption were carried out at our laboratory by mixing the new polyclonal antibody with different proteinaceous solutions to allow non-specific antibodies or antibodies with cross-reactivity to bind proteins in the solutions and precipitate. The new polyclonal antibody was mixed with 1) culture filtrates (3 mg/mL) from *M. bovis* BCG Copenhagen, a BCG sub-strain that lacks Region of Difference 2 and therefor does not express MPT64 protein, in ratio 1:1, or 2) BCG Copenhagen cell sonicate and antibody in ratio 1:5, or 3) homogenised non-TB lung and lymph node tissue sections (deparaffinised and hydrated) in PBS in ratio 1:1. The mixture was gently vortexed and left overnight at 4°C. The absorbed antibodies were then carefully pipetted off the precipitated antibody-antigen complexes and tested in IHC.

Protocol for preparation of homogenised tissue solutions

Formalin-fixed and paraffin-embedded non-TB lung and lymph node biopsies were selected for absorption experiments because the new polyclonal antibody showed a high degree of non-specific staining in these biopsies. Six 10 µm thick sections from each biopsy were placed in a tube, deparaffinized with xylene and rehydrated through decreasing grades of alcohol. After three minutes in distilled water, the tubes were centrifuged, the water pipetted off and 1 mL PBS was added to each tube. The tissue/PBS mixture was transferred to a lysing matrix tube A (MPbio.com) containing garnet matrix and ¼ ceramic sphere and homogenized for 40 seconds

by a Fastprep-24-5G homogenizer. The lysed tissue mixture was subsequently used directly for absorption experiments as described above.

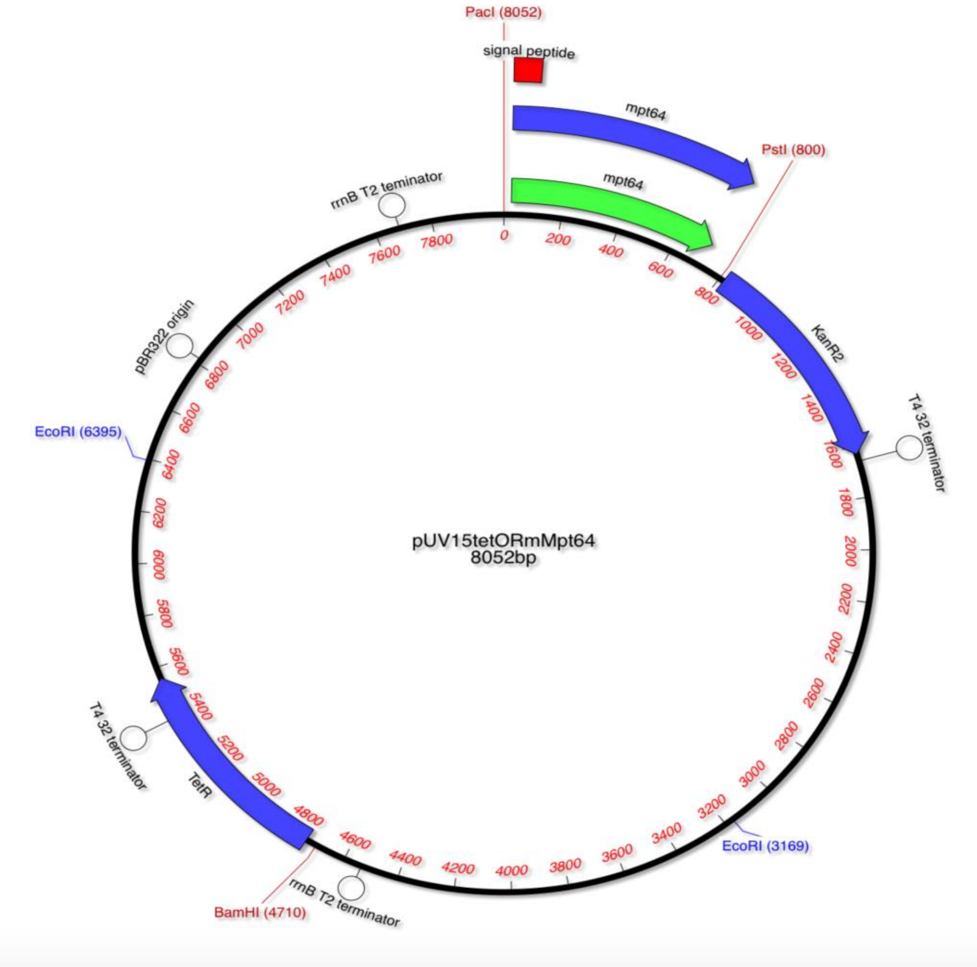

Figure S1. The completed vector construct, pUV15tetORmMpt64, expressing MPT64 protein. An 800 bp fragment containing the gene encoding the MPT64 protein, including its secretion signal sequence, was inserted between the PacI and PstI sites of the pUV15tetORm plasmid, a vector stable in *M. smegmatis* and *E. coli*.

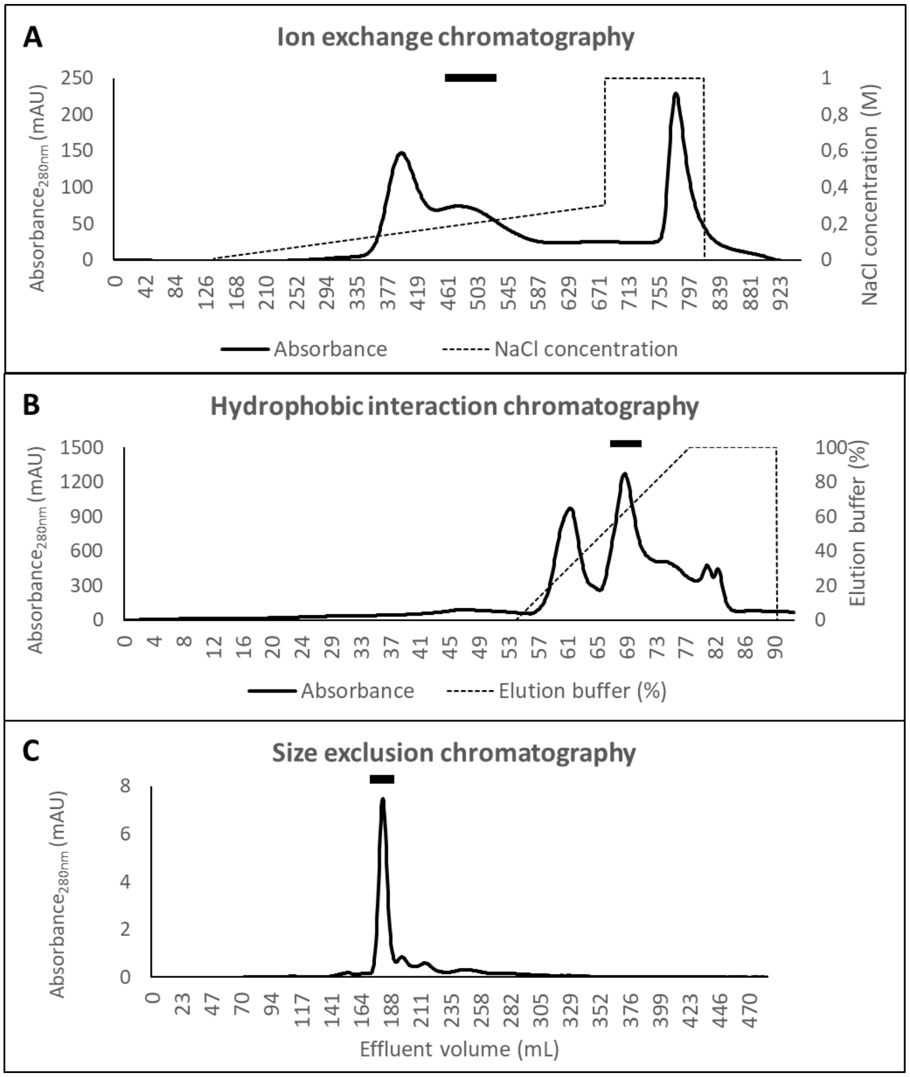

Figure S2. Chromatograms from the three-step chromatography purification strategy applied to purify native MPT64 protein. The elution pattern from the first step separation of culture filtrate proteins from M. bovis BCG Moreau by anion exchange chromatography on a Q Sepharose Fast Flow column is shown in (A). Fractions containing MPT64 were pooled and further purified in the intermediate hydrophobic interaction chromatography step on a Phenyl HP HiTrap column (B), before the final polishing step size exclusion chromatography (C) on a Superdex 75pg HiLoad column. Fractions of the eluate were consecutively collected during all runs and subjected to SDS-PAGE and Western blot to identify the MPT64 containing fractions. The bars in the chromatograms indicate the part of the eluate that contained MPT64.

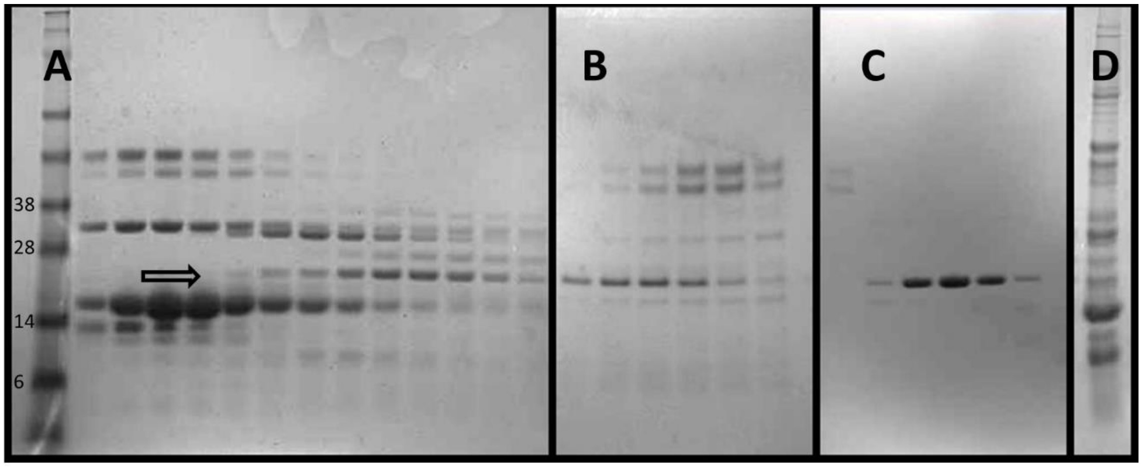

Figure S3. Overview of purity of the MPT64 containing fractions after each step of chromatography. The figure shows the purity of the fractions containing MPT64 protein in Coomassie-stained SDS-PAGE after (A) ion exchange chromatography, (B) hydrophobic interaction chromatography and (C) size exclusion chromatography. (D) shows concentrated culture filtrates from M. bovis BCG. Molecular mass is given for the bands in the standard (SeeBlue Plus2, Invitrogen, CA, USA) at the left in (A). The MPT64 band is located at approximately 24 kDa (arrow).
